# Evaluating Nitrogen Management Options for Reducing Nitrate Leaching from Northeast U.S. Pastures

**DOI:** 10.1100/tsw.2001.253

**Published:** 2001-10-09

**Authors:** William L. Stout, James E. Delahoy, Laurence D. Muller, Louis S. Saporito

**Affiliations:** The United States Department of Agriculture/Agricultural Research Service, University Park, PA 16802, USA

**Keywords:** water quality, intensive grazing, nitrate, nutrient management, agroecosystems, dairy cow nutrition

## Abstract

Substantial amounts of nitrate nitrogen NO_3_-N can leach from intensively grazed pasture in the northeast U.S. where there is about 30 cm of groundwater recharge, annually. Management options for reducing NO_3_-N leaching were evaluated for this environment using the Cornell Net Carbohydrate and Protein System Model and a recently developed nitrogen leaching index. Management options utilizing energy supplementation of grazing dairy cows could improve nitrogen efficiency within the cow, but would not necessarily reduce NO_3_-N leaching at the pasture scale if stocking rate was not controlled. The management option of using white clover to supply nitrogen to the pasture decreased NO_3_-N leaching, but produced less dry matter yield, which in turn reduced stocking rate. The economic returns of reducing NO_3_-N with these options need to be evaluated in light of milk prices and commodity and fertilizer nitrogen costs. At current prices and costs, the economic benefit from the energy supplementation options is substantial.

## INTRODUCTION

In recent years, there has been renewed interest in better utilizing grazing lands in the northeast U.S. Much of this renewed interest involves using the practice of management-intensive rotational grazing (MIRG) to increase the profitability of small- and medium-sized dairy farms common to this region. Economic studies have shown that MIRG can increase the profitability of these dairy farms by about $150 per cow per year. Much of this improved profitability is the result of reduced operating costs rather than increased milk production. In addition, many farmers report a better quality of life because of reduced workload in comparison to conventional confinement dairy farming; however, MIRG is not without problems, chiefly the increased potential for nitrate leaching.

Nitrate leaching from grazed grasslands is the result of nitrogen in pasture herbage being ingested, concentrated in urine and deposited in concentrated urine spots across the pasture landscape. The nitrogen application rate to the pasture in these concentrated urine spots is about 500–700 kg $ha^{-1}$, a rate much in excess of the amount that can be utilized by the impacted herbage in a growing season. Consequently, much of the excess nitrogen is subject to leaching below the root zone. This phenomenon was first documented by Ryden et al.[1] and has been further quantified by others in maritime climates such as the U.K.[2] and Europe[3]. Recent work in the humid continental climate of the northeast U.S. has documented nitrate leaching losses comparable to those reported in the U.K., New Zealand, and Europe[4,5]; however, documenting such losses is only the first step in developing management options so that both environmental and farm profitability goals can be met.

Cuttle and Scholefield[6] identified several management options to reduce nitrate leaching losses from intensively managed grasslands, the most obvious of which is to reduce fertilizer nitrogen inputs; however this option also reduces profitability unless lower production levels can be offset by lower production costs associated with grass/cloverpastures. Other management options for reducing $NO_3$-N leaching that were identified included modification of animal diets and more efficient use of animal manures and slurries. The objective of this paper is to use an animal nutrition model in combination with a leaching index to evaluate the effect of two management options (reducing fertilizer nitrogen input and modification of animal diets) on nitrogen leaching.

## METHODS AND MATERIALS

The animal nutrition model used was the Cornell Net Carbohydrate and Protein System (CNCPS)[7]. The CNCPS is a nutritional computer model that uses animal, environmental, management, and feed inputs to predict animal performance under differing nutritional situations. The model is based on a set of sub models including intake, feed evaluation, environmental, ruminal fermentation, intestinal digestion, absorption, tissue utilization, and excretion. The equations and validation of the CNCPS have been published[8,9,10,11,12,13,14,15]. Kolver[16,17] has recently validated the CNCPS for lactating dairy cows on pasture.

The nitrogen leaching index that relates stocking rate and $NO_3$-N leaching loss was that developed by Stout et al.[5]. This index contains four components: (1) background $NO_3$-N leaching loss from pasture not impacted by excreta; (2) $NO_3$-N leached from the daily urine excretions of a single animal; (3) cumulative seasonal stocking rate; and (4) a correction for multiple urine depositions with increased stocking rates[18,19] on the same site in the pasture. Components 1 and 2 were determined from a series of field studies with large drainage lysimeters in which $NO_3$-N leaching loss from different sward types and animal excreta were measured[5,20,21]. In addition, for Component 2, the volume and number of daily excretions, urine composition as a function of season, and the amount of time that the animals would be on pasture were also considered. Finally, for component 2, only the $NO_3$-N from urine is used for the projections in this study. This simplification is justified by the fact that the contribution of feces to leachate nitrogen over the three years of the study was less than 0.2% of the amount contributed by urine[20]. Component 3, stocking rate, was determined by dividing the total herbage on offer in the pasture[20,21] by the daily dry matter intake of that forage by the grazing animal. Component 4 increases $NO_3$-N leaching under multiple urine deposition areas as determined by projected stocking rate (Component 3) and a negative binomial distribution of urine deposition on pastures[18,19].

The objective of this paper was to use the CNCPS to determine the impact of three "typical" feeding scenarios for lactating dairy cows grazing pasture on nitrogen excretion. Nutritional composition of feeds and animal parameters for CNCPS inputs were based on previous grazing studies at Penn State[16,17]. Supplemental diets were based on the CNCPS feeds library. In scenario 1, a CNCPS simulation was run to determine the excretion of nitrogen for cows grazing all-grass pasture (Table 1). In scenario 2, a CNCPS simulation was run for cows fed grass pasture and supplemented with a high-protein energy feed. In scenario 3, a CNCPS simulation was run for cows fed grass pasture and supplemented with a low-protein energy feed. The rationale behind scenarios 2 and 3 is that the increased energy in the supplement increases milk production enabling the animal to route more of the excess protein in pasture into milk nitrogen rather than urinary nitrogen. In scenarios 2 and 3 feed intake was set higher because of overall increases in intake seen when pasture is supplemented with energy/protein feeds composed primarily of corn and soybean[22]. The urinary nitrogen output from the CNCPS simulations were then used in the nitrogen index to determine $NO_3$-N concentrations in leachate for the 'all-pasture' scenario in a nitrogen-fertilized orchardgrass pasture and an orchardgrass/white clover pasture, and for the 'low-protein' scenario in a nitrogen fertilized orchardgrass pasture. The effect of the three management scenarios on $NO_3$-N leaching was evaluated at 60% utilization of the total annual available forage harvested at a 7.5 cm stubble height[21].

**TABLE 1**
**Summary of Assumptions for All-Pasture, High-Protein, and Low-Protein Feed Scenarios Developed with the CNCPS Model**

| | All-Pasture | High-Protein | Low-Protein |
|---|---|---|---|
| Milk Yield, kg day$^{-1}$ cow$^{-1}$ | 18 | 27 | 27 |
| Milk Protein, % | 3.1 | 3.3 | 3.3 |
| Pasture Intake, kg day$^{-1}$ cow$^{-1}$ | 15.9 | 12.5 | 12.5 |
| Supplement Intake, kg day$^{-1}$ cow$^{-1}$ | 0 | 6.8 | 6.8 |
| Pasture Crude Protein, % | 21* | 21 | 21 |
| Supplement Crude Protein, % | – | 18 | 12.5 |
| Energy, Mcal kg$^{-1}$ | 1.6 | 1.7 | 1.7 |

* Pasture crude protein assumed to be 76% rumen degradable for both pasture types.

## RESULTS AND DISCUSSION

The high and low protein supplementation scenarios increased the daily nitrogen intake over the all-pasture scenario by 0.08 and 0.02 kg day$^{-1}$, respectively (Table 2). Consequently, fecal nitrogen for the two supplementation scenarios was also increased by 0.02 kg day$^{-1}$; however, only the high-protein supplementation scenario increased urinary nitrogen over that of the all-pasture scenario. The low-protein scenario decreased urinary nitrogen with respect to the all-pasture scenario by 0.04 kg N day$^{-1}$ (Table 2). Both supplementation scenarios increased milk nitrogen (N), largely because of the increased energy in the diet and subsequent increase in milk production (Table 1). Because of the increased milk production both supplementation scenarios increased nitrogen efficiency [(milk N/ N intake) × 100]; however, from the water-quality standpoint, the high-nitrogen supplementation scenario would not be desirable because of increased urinary nitrogen. Urinary nitrogen is the main mode of increased nitrogen leaching loss under pasture.

Projections of the individual cow nitrogen balances for the all-pasture and low-protein supplementation scenarios are presented in Fig. 1. For these projections, we assumed 60% utilization of a nitrogen-fertilized (250 kg ha$^{-1}$) orchardgrass pasture. Although the low-protein supplementation scenario decreased urinary nitrogen with respect to the all-pasture scenario for an individual cow (Table 1), it had no appreciable effect on the $NO_3$-N concentration in the estimated leachate below a pasture at 60% utilization of available forage (Fig. 1). This is because the supplementation scenario allows a higher stocking rate ~640 au days ha$^{-1}$) than the all pasture scenario (~480 au days ha$^{-1}$); however, if stocking rate were set the same as the stocking rate as 60% utilization of the all-pasture scenario, the low-supplementation scenario would result in a decrease of about 2 mg l$^{-1}$ in the estimated $NO_3$-N concentration in the leachate below the root zone.

At 60% utilization of available forage, the estimated $NO_3$-N concentration in the leachate below the root zone of orchardgrass/white clover pasture (~10 mg l$^{-1}$) was about 80% lower than that of the All-Pasture scenario (~18 mg l$^{-1}$) at the same level of forage utilization (Fig. 1). There were two reasons for this lowered level of leachate. First, estimated $NO_3$-N concentration at 0.0 au days ha$^{-1}$ was lower for the orchardgrass/white clover pasture[21].

**TABLE 2**
**Daily Nitrogen Balance for an Individual Cow on All-Pasture, High-Protein, and Low-Protein Feed Scenarios Developed with the CNCPS model**

| | All-Pasture | High-Protein | Low-Protein |
|---|---|---|---|
| N intake, kg day$^{-1}$ | 0.53 | 0.61 | 0.55 |
| Fecal N, kg day$^{-1}$ | 0.16 | 0.18 | 0.18 |
| Urinary N, kg day$^{-1}$ | 0.22 | 0.23 | 0.18 |
| Milk N, kg day$^{-1}$ | 0.095 | 0.15 | 0.15 |
| N efficiency, % | 17.9 | 24.5 | 27.2 |

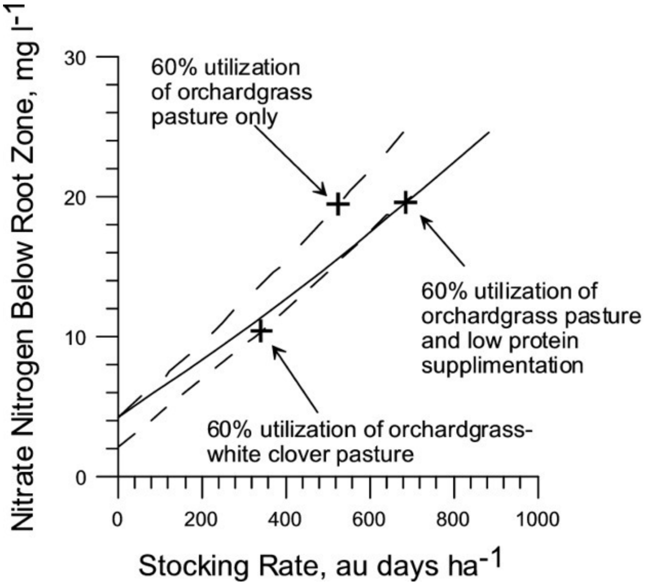

**FIGURE 1.** Relationship between $NO_3$-N in leachate beneath pasture and stocking rate.

Second, lower dry-matter yields of the orchardgrass/white clover pasture (~7.7 Mg $ha^{-1}$) compared to that of the nitrogen-fertilized orchardgrass pasture (~10.5 Mg $ha^{-1}$)[20] reduced the stocking rate at the 60% utilization level.

Utilizing supplementation to reduce $NO_3$-N leaching loss from nitrogen fertilized pasture would only be successful if stocking rate is limited to that of an All-Pasture scenario. Even producing the grain locally for the supplementation may not improve the situation because $NO_3$-N concentration from leachate under well-managed corn produced in this region can range from 12–20 mg $l^{-1}$[23]. This means that nitrogen leaching from the farm or watershed would be the same regardless of whether it was utilized for pasture or corn production.

Importing supplement to the farm would further exacerbate nitrate leaching because it would result in increased nutrients on the farm and would support a higher stocking rate. Importing nutrients to the farm is economically attractive because of the current low price of corn.

The scenario of grazing an orchardgrass/white clover pasture at 60% utilization is the only evaluated scenario that resulted in an estimated $NO_3$-Nn concentration in the leachate that was close to the U.S. Environmental Protection Agency's drinking water standard of 10 mg $l^{-1}$[24] of $NO_3$-N. Whether reduced production costs can offset the decreased stocking rate and subsequent milk production levels inherent in such a scenario depends on economic factors not evaluated here.

## CONCLUSIONS

Stocking rate is the major factor controlling the concentration of $NO_3$-N in leachate below the root zone of nitrogen-fertilized, intensively grazed orchardgrass pastures. Supplementation of animals on pasture with energy supplements can improve nitrogen efficiency within the animal but not necessarily reduce nitrogen leaching from pastures, unless stocking rate is controlled. Uncontrolled stocking rates could result in intensively grazed pastures becoming essentially well-grassed feedlots that are not necessarily as environmentally benign as a well-designed and well-managed confinement feeding system. Relative to nitrogen fertilization of pastures, utilization of white clover to supply nitrogen to the pastures can reduce $NO_3$-N leaching from pastures through its subsequent reduction in stocking rate.

| | | |
|---|---|---|
| Received: | July | 16, 2001 |
| Revised: | September | 9, 2001 |
| Accepted: | September | 9, 2001 |
| Published: | October | 9, 2001 |
